# Are poor set-shifting abilities associated with a higher frequency of body checking in anorexia nervosa?

**DOI:** 10.1186/s40337-015-0053-3

**Published:** 2015-04-12

**Authors:** Maria Øverås, Hilde Kapstad, Cathrine Brunborg, Nils Inge Landrø, Bryan Lask

**Affiliations:** Regional Department for Eating Disorders (RASP), Division of Mental Health and Addiction, Oslo University Hospital, Ullevål, Pb 4950 Nydalen, 0424 Oslo, Norway; Unit of Biostatistics and Epidemiology, Oslo University Hospital, Ullevål, Pb 4950 Nydalen, 0424 Oslo, Norway; Department of Psychology, Clinical Neuroscience Research Group, Box 1094, , Blindern, 0317 Oslo, Norway; Care UK, London, UK; Great Ormond Street Hospital for Children, London, WC1 N 3JH UK

**Keywords:** Anorexia nervosa, Eating disorders, Body checking, Set-shifting, Cognitive flexibility

## Abstract

**Background:**

The rigid and obsessional features of anorexia nervosa (AN) have led researchers to explore possible underlying neuropsychological difficulties. Numerous studies have demonstrated poorer set-shifting in patients with AN. However, due to a paucity of research on the connection between neuropsychological difficulties and the clinical features of AN, the link remains hypothetical. The main objective of this study was to explore the association between set-shifting and body checking.

**Methods:**

The sample consisted of 30 females diagnosed with AN and 45 healthy females. Set-shifting was assessed using the Wisconsin Card Sorting Test (WCST) and frequency of body checking was assessed using the Body Checking Questionnaire (BCQ).

**Results:**

The analysis showed no significant correlations between any of the WCST scores and the BCQ.

**Conclusion:**

The results suggest that there is no association between set-shifting difficulties and frequency of body checking among patients with AN. An alternative explanation could be that the neuropsychological measure included in this study is not sensitive to the set-shifting difficulties observed in clinical settings. We recommend that future studies include more ecologically valid measures of set-shifting in addition to standard neuropsychological tests.

## Background

Rigidity and obsessionality are common features in patients with anorexia nervosa (AN) [1]. The rigid and obsessive behaviour are often focused on eating, exercise, weight and shape, and include behavioural aspects of AN such as body checking [2].

Body checking is the compulsive checking of one’s body, with the aim to monitor shape and weight changes [2]. It includes behaviours such as ritualistic weighing, feeling for bone protrusion, pinching for fatness, or trying on special clothing [3,4]. Several studies have demonstrated significantly more body checking in patients with AN compared to healthy controls [2,5]. Moreover, body checking is highly associated with the degree of eating disorder psychopathology [5].

Patients describe body checking as safety behaviour, aiming to reduce anxiety about body weight and shape [6]. Paradoxically, the long-term effect is often the opposite, with increased attention towards body weight and shape [4,6,7], more body dissatisfaction [8], more restrictive eating [5] and lower quality of life [9]. In sum, body checking seems to be both a consequence of AN and a maintaining factor of the disorder. Understanding what drives body checking in patients with AN is therefore highly relevant for designing efficient treatment interventions.

The rigid and obsessive features of AN have led researchers to question whether there might be underlying neuropsychological difficulties in such areas as set-shifting [10]. Set-shifting refers to the ability to switch focus back and forwards between tasks, operations or mental sets [11]. It is suggested that set-shifting difficulties in patients with AN could represent both a risk factor for developing AN and a maintaining factor [12]. Several studies have explored set-shifting in patients with anorexia nervosa. The predominant finding is that patients with AN commonly manifest difficulties with set-shifting tasks [13-15], although there are a few studies reporting no differences between patients with AN and healthy controls [16,17]. Furthermore, most studies of set-shifting in AN are carried out with adult participants. Studies including younger participants with AN have indicated that there might be less set-shifting difficulties in this group e.g. [18].

It is unclear whether set-shifting difficulties among patients with AN are a pre-existing trait contributing to the development of an eating disorder, or a consequence of other factors such as extreme underweight or comorbid psychiatric conditions [10,19,20]. Several studies of set-shifting in patients with AN have controlled for BMI (body mass index) to assess the impact of low weight. Despite some conflicting findings, the majority of studies report that weight status does not significantly affect set-shifting abilities in patients with AN [10,19-21]. In addition, several studies have demonstrated set-shifting difficulties in weight-recovered AN-patients [13,20,22-24], and in healthy sisters of patients with AN [20,25], also indicating that low weight is not a defining factor.

With regard to the relative contribution of comorbid conditions, most studies have focused on depression and/or anxiety. The evidence so far has been inconclusive. Giel et al. [19] found that patients with unipolar depression performed significantly poorer on set-shifting tasks compared to patients with AN. Further, they found that performance on the set-shifting task for both groups was negatively correlated with degree of depressive symptoms [19]. In contrast, other studies report no significant relationship between depression and anxiety symptoms on set-shifting performance in patients with AN [10,20].

Research exploring set-shifting abilities in patients with AN has included a variety of different neuropsychological tests. One of the most commonly used tests is the Wisconsin Card Sorting Test (WCST) [26]. The WCST produces a variety of scores reflecting different aspects of the participants’ performance. Although all scores are assumed to reflect set-shifting abilities to some degree, the perseveration scores are often referred to as the main indicators of set-shifting performance [26] (for a more thorough description of WCST, see the method section below). A large number of studies have explored performance on the WCST among patients with AN compared to healthy controls. A few studies reported no differences between patients and controls in WCST performance [17]. However, several studies have reported significantly poorer WCST performance in patients with AN compared to healthy controls, including fewer *total correct* [10,23,27], more *perseverative errors* [10,20,21,23,24,27], more *non-perseverative errors* [23,27], and fewer *categories completed* [20,21,23,28].

To summarize, numorous studies have indicated poorer performance on set-shifting tasks in patients with AN compared to healthy controls. Albeit there has been surprisingly little research exploring the neuropsychological profiles’ connection to the actual cognitive and behavioural features of AN. Findings from the few studies exploring the connection between neuropsychological profile and clinical features of AN are conflicting. For example, in a study by Talbot, Hay, Buckett and Touyz [29] they did not find any association between set-shifting performance and clinical features of AN (measured by EDE-Q). It should be noted that in this study, they did not find any difference in set-shifting performance between acute ill patients with AN and healthy controls [29], which could have contributed to the lack of association between clinical features and set-shifting. In contrast in Harrison, Tchanturia, Naumann & Treasure [30] found that a subgroup of AN-patients with higher degrees of set-shifting difficulties was associated with a more chronic form of illness.

Body checking is a central feature of AN, assumed to contribute to the maintenance of the disorder. Hence, it is of great importance to understand the underlying factors contributing to this behaviour. Body checking resembles the checking behaviour seen in patients with obsessive-compulsive disorder (OCD) [31]. Both types of checking seem to involve difficulties deflecting from an established course of thoughts or action (perseveration) [32]. That is, when a patient with AN repeatedly checks her stomach in response to worries about becoming fat, the assumption made by Harvey [32] is that she repeats the checking because she is unable to shift her mindset about being fat despite the information she gets from the checking. It has been suggested that this type of perseveration is a result of impaired set-shifting abilities [26,32]. Several studies have confirmed this link between compulsive checking and set-shifting difficulties among patients with OCD [32-34] and in nonclinical samples [35]. However, no studies to our knowledge have explored the association between set-shifting difficulties and compulsive body checking in patients with AN.

The main aim of this study was to explore any association between set-shifting and frequency of body checking in patients with AN and healthy controls. Secondly we wanted to see if we could replicate findings from previous studies of significant difference in set-shifting abilities between patients and controls. Based on previous findings, our hypothesis was that patients with AN would perform significantly less well on the set-shifting task, compared to healthy controls. Furthermore, we hypothesised that poorer set-shifting performance would be associated with higher degrees of body checking.

## Methods

### Sample

The sample consisted of 30 females diagnosed with AN – restrictive subtype and 45 healthy females aged between 14 and 27 years. The patients were recruited from five different units specializing in the treatment of eating disorders in Norway. Diagnoses were made based on the diagnostic criteria from the *Diagnostic and Statistical Manual of Mental Disorders, 4th edition* (APA, 2000), using the diagnostic items of the *Eating Disorder Examination* (EDE OD12) [36]. The interviews were performed and rated by trained interviewers. Amenorrhea was not included as a diagnostic criterion, as we anticipated its removal from DSM-5 [37]. Also, four patients were included who initially fulfilled the criteria for AN, but who had just exceeded the weight-threshold at the time of assessment, due to weight restoration during hospital admission.

The controls were recruited from local schools and universities in Oslo, Norway. The recruitment was initated by giving information about the study at lectures, asking students to write down contact information before leaving if they were interpreted in participation. Controls were screened with the *Eating Disorder Examination – Questionnaire* (EDE-Q) [38] to ensure that they did not have an eating disorder. Two controls were excluded due to self-reports of a current eating disorder. An EDE-Q cut-off score of 2.77 was used for the control group, based on a large normative study by Mond, Hay, Rodgers and Owen [39]. Five controls were excluded due to a global EDE-Q score above the cut off.

The study received approval from the Norwegian Regional Ethical Committee for Medical Research, and informed consent was obtained from all the participants.

### Measures

**Eating Disorder Examination - Questionnaire version (EDE-Q)** to assess eating disorder psychopathology [38]. The EDE-Q was adapted from the clinical interview Eating Disorder Examination (EDE) [38], and later translated into Norwegian. Both the original form and the Norwegian translation have demonstrated good psychometric properties [38,40].

**Body Checking Questionnaire (BCQ)** to assess body checking behaviour [2]. The questionnaire consists of 23 statements about body checking behaviour (e.g. “I pinch my stomach to measure fatness”), which the respondent rates on a Likert scale, from one to five with higher scores indicating more body checking (1 = “never” and 5 = “very often”). The score included in the analysis are the total score, which is calculated by summarizing the scores on each item. Both the original version and the Norwegian translation have demonstrated good psychometric properties [2,3].

**State-Trait Anxiety Inventory (STAI)** to assess anxiety [41]. Contrary to other measures of anxiety, the STAI distinguishes between current anxiety (state-anxiety) and general level of anxiety (trait-anxiety). In this study, only the trait-anxiety scale is included. Both the original version of STAI and the Norwegian translation have demonstrated good psychometric properties [41,42].

**Beck Depression Inventory (BDI-II)** to assess severity of depressive symptoms [43]. Both the original version of BDI-II and the Norwegian translation have demonstrated good psychometric properties [43,44].

**Wisconsin Card Sorting Test (WCST-64) computer version 2: Research edition** to assess set-shifting [26]. WCST is a well-established assessment tool with good psychometric properties [26]. There are convincing data from several studies to support the comparability between the standard version and the 64-version of WCST [45]. Although all scores are assumed to reflect set-shifting abilities to some degree, the perseveration scores are often referred to as the main indicators of set-shifting performance. Total errors, perseverative scores and conceptual level responses were converted into t-scores using age-corrected normative data (age-corrected t-scores for participants below 20 years, U.S. census age-matched t-scores for participants above 20 years). The remaining sub-scales are reported as raw scores.

During the test, participants sit in front of a computer screen. On the screen, there are four stimulus cards arranged horizontally and a deck of 64 response cards placed below the stimulus cards. The four stimulus cards reflect three stimulus parameters: form (crosses, circles, triangles or stars), colour (red, blue, yellow, or green) and number (one, two, three, or four figures). The participant is asked to sort the response cards one by one, by placing it beneath one of the four stimulus cards, wherever she thinks it should go. The matching can be done based on any of the three stimuli parameters (form, colour or number). After each response, the computer informs the participant whether she was correct or incorrect. In effect, the participant must infer the correct sorting principle on a trial and error basis. After each ten consecutive correct responses, the sorting principle is switched without warning and the participant must abandon the previous principle and deduce the new one. Scores are derived based on how well the participants adapt to these changes in sorting principles during the test. The test has no time limit, although estimated administration time is 10–15 minutes.

The following scores are derived: total correct (all correct responses summarized), perseverative responses (number of incorrect responses that would have been correct following the previous sorting principle), perseverative errors (number of incorrect responses where the participants have used the same sorting principle as their previous choice, i.e. failed to correct the sorting principle based on feedback), non-perseverative errors (all errors that are not perseverative), conceptual level responses (percentage of consecutive correct responses occurring in runs of three or more), categories completed (number of runs of ten correct responses), failure to maintain set (the number of times five or more correct responses occurred without completing the category), and trials to complete first category (the number of trials needed to achieve the first ten consecutive correct responses) [26].

**Matrix reasoning and Vocabulary** are neuropsychological tests from the Wechsler’s Intelligence Scale batteries (WAIS-III/WISC-III) [46,47]. Matrix reasoning and Vocabulary were included to provide an estimate of performance and verbal IQ respectively, and to ensure that patients and controls did not differ significantly on general cognitive abilities. The scores reported are scaled scores.

### Weight for height ratio

Children and adolescents have far greater variability in weight than adults, depending on variables such as height, age and gender. For this reason, we used the weight for height ratio, a calculation of body mass adjusted for height, gender and age [48], as an alternative to BMI. The weight for height ratio is reported as percentage of expected weight for gender, age and height.

The patients’ weights and heights were collected by clinical staff and reported to the researchers with the patients’ consent. For all patients, the weight included was recorded within one week from the neuropsychological assessment. In the control group, the researchers measured weight and height as part of the assessment.

### Procedure

The data collection was conducted in one scope for the control participants. For patients, the data collection was divided between two separate days to minimize possible effects of reduced concentration. All tests and questionnaires were administered within two weeks.

### Statistical analysis

Sample characteristics are presented as means with standard deviation (SD), medians with 95% confidence intervals (CI) or proportions. Differences in continuous variables between patients and controls were tested with Student t-tests for normally distributed data and Mann – Whitney U test for non-normally distributed data. Pearson or Spearman’s correlation coefficient was used to analyze the association between body checking and WCST, or between other continuous variables when appropriate. Adjustment for multiple confounding variables was made using linear regression analysis with a manual backward procedure, when studying the association between patients vs. controls as exposition and WCST as outcome. All statistical analyses were done using the PASW Statistics software version 18.0 (IBM SPSS Inc., Chicago, IL) and a significance level of 5% was used.

### Sample size

A de facto power analysis was performed, using WCST total correct raw score as the primary endpoint in this estimation. In our study, the estimated mean difference of WCST total correct raw score was 4.6 with a standard deviation of 8.2 in a sample of 30 cases and 45 controls. Thus, with a type I error of 5%, we would be able to reject the null hypothesis that the population means of the case and control groups are equal with a power of 70%.

## Results

### Sample characteristics

The sample characteristics are summarized in Table 1. The groups differed significantly on all parameters except age and performance and verbal IQ (Matrix reasoning and Vocabulary).

**Table 1:** **Sample characteristics**

	**Patients (n = 30)**	**Controls (n = 45)**	**p-value**
Age – m (sd)	19.07 (2.96)	18.31 (3.14)	.29
Weight for height – m (sd)	77.53 (7.48)	101.29 (13.00)	<.001
EDE-Q total – m (sd)	3.67 (0.98)	0.79 (0.59)	<.001
BCQ – m (sd)	67.64 (21.22)	40.84 (11.08)	<.001
STAI-trait – m (sd)	62.40 (7.78)	36.88 (7.88)	<.001
BDI-II – m (sd)	33.62 (11.10)	5.98 (4.45)	<.001
Matrix reasoning – m (sd)^#^	11.00 (2.83)	11.89 (2.01)	.16
Vocabulary – m (sd)^#^	10.31 (1.82)	10.39 (2.31)	.87

*Abbreviations*: *EDE-Q* (Eating Disorder Examination –Questionnaire version), *BCQ* (Body Checking Questionnaire), *STAI-trait* (State Trait Anxiety Inventory – trait section), *BDI-II* (Beck Depression Inventory).

# 7 or 8 missing values (if not specified, missing < 10%).

### Set-shifting: patients vs. controls

As shown in Table 2, patients performed significantly poorer than healthy controls on several of the subscales of WCST, including *total correct*¸ *non-perseverative errors*, *conceptual level responses* and *categories completed.* The effect sizes were between medium and large. It should be noted that all the scores, both for patients and controls, were within one standard deviation from the normative mean.

**Table 2:** **Set-shifting: Wisconsin Card Sorting Task**

	**Patients (n = 30)**	**Controls (n =45)**	**p-value**	**Cohen’s d**
Total correct, m(sd)*	46.47 (10.05)	51.11 (6.13)	<.05	0.56
Perseverative responses, m(sd)	50.93 (11.27)	55.53 (9.98)	.07	0.43
Perseverative errors, m(sd)	51.07 (11.10)	55.11 (9.51)	.10	0.39
Nonperseverative errors, m(sd)	47.27 (10.21)	53.67 (7.32)	<.01	0.72
Conceptual level response, m(sd)	48.63 (10.81)	55.49 (7.82)	<.01	0.73
Categories completed, m(sd)*	3.30 (1.42)	3.91 (1.01)	<.05	0.50
Failure to maintain set, m(sd)*	0.40 (0.62)	0.36 (0.57)	.80	0.07
Trials to complete 1st category, median (95% CI)**	12 (11–13)	11 (11–12)	.15	540.0/1.439***

*Abbreviations*: *WCST* (Wisconsin Card Sorting Test).

When nothing else is noted, the numbers reported are t-scores. All t-scores are age-corrected.

*Raw score.

**Mann- Whitney U Test.

***The Mann–Whitney U test statistic and Z-score: U/Z.

### Controlling for possible confounders

To identify possible confounders, we explored variables that have been shown to affect performance on WCST in previous studies (weight for height ratio, depression and anxiety). The IQ-indicators (Matrix reasoning and Vocabulary) were excluded as possible confounders, since they did not differ significantly between patients and controls. Of the three other variables, only those with significant relationships with both exposure (patients vs. controls) and outcome (WCST) were considered as possible confounders and included in the analysis. As indicated in Table 1, all three variables differed significantly between patients and controls. With regard to WCST, weight for height ratio was significantly correlated with *non-perseverative errors* (r = 0.24, p = 0.04) and *conceptual level responses* (r = 0.28, p = 0.01) of the WCST. Anxiety was significantly correlated with *non-perseverative errors* (r = −0.28, p = 0.01) and *conceptual level responses* (r = −0.27, p = 0.02), and depression only with *conceptual level responses* (r = −0.29, p = 0.01).

Next we performed a multivariate linear regression analysis to adjust for the identified possible confounders, with each of the relevant WCST scores. After controlling for weight for height ratio, the difference between patients and controls became non-significant for *conceptual level responses* (β_adj_ = 5.65, 95% CI: −0.69 – 11.99, p = 0.08, r^2^ = 0.12). None of the other confounders had any significant effect on the WCST results for patients vs. controls.

### Correlations between body checking and set-shifting

When exploring the association between set-shifting difficulties and body checking, analyses were conducted separately for patients and controls. The results showed that body checking (BCQ) was not significantly correlated with any of the set-shifting measures (WCST) in either group. Results are summarized in Table 3.

**Table 3:** **Associations between body checking and set-shifting**

	**Patients (n = 30)**		**Controls (n = 45)**	
	**Body checking questionnaire**		**Body checking questionnaire**	
	**r**	**p-value**	**r**	**p-value**
WCST				
- Total correct, raw score	-.25	.19	-.05	.77
- Perseverative responses	-.08	.67	.02	.89
- Perseverative errors	-.13	.50	-.01	.97
- Nonperseverative errors	-.03	.89	-.10	.50
- Conceptual level responses	-.17	.37	-.08	.61
- Categories completed	-.23	.22	-.04	.78
- Failure to maintain set	.05	.78	.20	.19
- Trials to complete 1st category	.24	.21	.21	.18

*Abbreviations*: *WCST* (Wisconsin Card Sorting Test).

## Discussion

The main aim of this study was to explore any association between set-shifting and frequency of body checking in patients with AN and healthy controls. Secondly we wanted to see if we could replicate findings from previous studies of significant difference in set-shifting abilities between patients and controls. First, we explored set-shifting differences between patients with AN and healthy controls. The analysis showed significant differences between groups on several subscales of the WCST. However, there were no significant differences on the perseverative subscales, which are assumed to be the strongest indicators of set-shifting difficulties. This finding is in contrast to reports from several other studies [10,20,21,23,27], and could reflect a selection bias or methodological differences between studies. For example, most previous studies have used the standard version of WCST, whereas we used the short form (WCST-64) to minimize any potential concentration difficulties due to low weight in the patient group. There are convincing data from several studies to support the comparability between the standard version and the 64-version of WCST [45]. As comparability between WCST versions has not been directly explored in an eating disorder population, we cannot rule out the possibility that this methodological difference affected our results.

Next, we explored the association between set-shifting and body checking. The analysis showed no significant correlation between the two. Thus, our hypothesis of a link between set-shifting difficulties and body checking was not supported by the data. Accordingly, we have to consider that this link might not exist. On the other hand, there are several possible explanations that should be considered prior to drawing conclusions on the matter. For example it is feasible that the association between set-shifting and body checking would be stronger if the patients in our sample had more perseverative difficulties, as has been found in several other studies. Further research would be needed to explore this possibility.

A second possible explanation lies in the assumptions about how set-shifting and body checking are connected. The assumption underlying this study was that greater set-shifting difficulties result in more perseveration in daily life, which would be reflected by more body checking behaviour. However, if set-shifting difficulties do not contribute directly to the amount of body checking, but rather represent a trait that interferes with the reduction of body checking during treatment, this might not be apparent in a correlational analysis. Studies suggesting that set-shifting difficulties in pateints with AN persist during weight gain e.g. [29] could be supportive of this hypothesis. Future studies on the treatment of body checking behaviour could potentially explore this hypothesis by including measures of set-shifting at pre-treatment to determine effects on prognosis, course, and outcome.

Third, the lack of association between set-shifting and body checking could be a result of the WCST not being sensitive to set-shifting problems observed in everyday life of patients with AN. In a study by Lounes, Khan and Tchanturia [14], a neuropsychological test (Brixton) and a self-report measure (Cognitive Flexibility Scale) were compared, both aiming to assess set-shifting difficulties. Even though patients with AN showed significantly lower scores compared to healthy controls on both measures, there was no significant association between the scores on the two measures in any of the groups [14]. This could indicate that the set-shifting problems measured by neuropsychological tests are qualitatively different from what we interpret as set-shifting difficulties in the everyday lives of our patients. We recommend that further research aims to explore this possibility, for example by including more ecologically valid measures of set-shifting, such as the Cognitive Flexibility Scale, in addition to standard neuropsychological tests.

Another possible explanation might relate to the fact that performance on most neuropsychological tests, including the WCST, relies on more than one cognitive ability. In other words, we cannot exclude the possibility that the our results might be influenced by other functions than set-shifting abilities. To address this challenge, future studies of the association between set-shifting and clinical features of AN could benefit from including a larger battery of tests measuring set-shifting abilities.

In line with several previous studies, we found that patients with AN performed less well on several WCST subscales, compared to healthy controls. The difference was significant for four out of eight WCST subscales and the effect sizes were medium to large. However, the difference between groups did not reach significance for the two WCST scores assumed to be the strongest indicators of set-shifting abilities (*perseverative responses* and *perseverative errors*). It is possible that differences might become significant with a larger sample, as we found a non-significant trend in this direction. To explore this possibility, post hoc power calculation for the perseverative responses was performed. The analysis confirmed that the power for these particular sub-scores were in fact lower (power: 44%, mean diff: 4.6, estimated sd:10.6) as compared to the WCST total score (70%). It should also be noted that our sample included a mix of younger and older patients (range 14–27 years). As studies indicate less difficulty with set-shifting in younger patients with AN [18] we cannot exclude the possibility that this might have affected our findings. In any case, the fact that all WCST scores of both patients and controls were within one standard deviation from the normative mean raises the question of whether these results, although statistically significant, are clinically significant. In other words, it might be that set-shifting scores so close to the normative mean would not have any observable effects on everyday life for patients with AN.

It should be noted that the comparisons between patients and controls should be interpreted with caution, since the large number of tests performed increases the likelihood of one or more false positives. Nevertheless, we have chosen not to adjust for multiple comparisons as our study already is underpowered and correction for type I errors cannot decrease without the possibility of inflating type II errors [49,50].

To make sure that confounding variables identified in previous studies did not account for the differences between patients and controls, we adjusted for several possible confounders, including weight for height ratio, anxiety and depression. When controlling for weight for height, the difference between patients and controls on *conceptual level responses* became non-significant. None of the other confounders contributed significantly to the model. Given that low weight can affect concentration [1] it is possible that *conceptual level responses* are particularly vulnerable to concentration difficulties. Obviously, this is highly speculative and no conclusion can be drawn based on the present data.

## Conclusion

Many studies have reported findings of neuropsychological difficulties in patients with AN. However there has been surprisingly little research exploring the neuropsychological profiles’ connection to the actual cognitive and behavioural features of AN. After all, the main significance of any neuropsychological differences between patients with AN and healthy controls is its potential clinical and preventative significance.

This study explored the association between body checking and set-shifting difficulties in patients with AN and healthy controls. The analysis showed no significant association between the two constructs. This may be a genuine finding but it is possible that methodological issues, discussed above, could account for the lack of association and these should be explored empirically before any final conclusions are reached.

## Abbreviations

AN: Anorexia nervosa
WCST: Wisconsin card sorting test
BCQ: Body checking questionnaire
OCD: Obsessive compulsive disorder
EDE-Q: Eating disorder examination – questionnaire version
STAI: State-trait anxiety inventory
BDI: Beck depression inventory
BMI: Body mass index
SD: Standard deviation
CI: Confidence interval
